# Factors Associated with Short-Term Surgical Outcomes Among Women Presenting with Pelvic Peritonitis at Bugando Medical Centre Mwanza, Tanzania

**DOI:** 10.24248/eahrj.v7i2.726

**Published:** 2023-11-30

**Authors:** Hija Hamadi, Happiness Mbena, Richard F. Kiritta, Oscar Ottoman, Vitus Silago, Mariam M. Mirambo, Stephen E. Mshana

**Affiliations:** a Department of Obstetrics and Gynecology, Weill Bugando School of Medicine, Catholic University of Health and Allied Sciences (CUHAS), Mwanza, Tanzania; b Department of Pathology, Weill Bugando School of Medicine, Catholic University of Health and Allied Sciences (CUHAS), Mwanza, Tanzania; c Department of Microbiology and Immunology, Weill Bugando School of Medicine, Catholic University of Health and Allied Sciences (CUHAS), Mwanza, Tanzania

## Abstract

**Background:**

Pelvic peritonitis is a rarely reported pathological condition in literature and emergency laparotomies are a common surgical procedure performed for these conditions in developing countries. This study was designed to investigate factors that are associated with short-term surgical outcomes among women with pelvic peritonitis.

**Methods:**

The study included retrospective and prospective data obtained between November 2021 and May 2022 from Bugando Medical Centre (BMC). Standardized data collection tool was used to capture clinical, surgical and laboratory data. Descriptive data analysis was done using STATA version 13.

**Results:**

A total of 101 participants were recruited; 22 prospectively and 79 retrospectively. The median age was 29[Interquartile range (IQR) 24 to 35] years. Majority (76.2%) of participants were referred patients. Obstetric related peritonitis 82.2% (83/101) was the most frequently detected with a necrotic and or dehiscent lower uterine segment (LUS) that necessitated a subtotal abdominal hysterectomy (STAH) being the commonest (40.8%) intraoperative finding. Postoperative complications were reported in 36.7% (36/98) and were significantly associated with pulse rates >100b/min (*P=.041*) and platelets <150 × 10^3^ mm^3^ (*P=.049*). The median length of hospital stay was 19[IQR: 7 to 35] days and temperature >37.5°C (aOR=5.08, 95% CI 1.23–20.97, P=.025) independently predicted prolonged hospital stay. Death occurred in 9.2% of patients and having ASA score of 5 (*P=.045*) was associated with death. Multi-drug resistant (MDR) gram-negative bacteria (85.2%) were the predominant pathogens causing pelvic peritonitis.

**Conclusion:**

A significant proportion of patients with pelvic peritonitis and deranged sepsis markers develop short-term surgical complications with a significant number of those with fever stay more than 14 days. There is a need of timely complete sepsis work up of the patients with pelvic peritonitis to ensure appropriate management is instituted to prevent associated morbidity and mortality.

## INTRODUCTION

Peritonitis, defined as an inflammation of the peritoneum, is a life threatening condition, which requires urgent surgical attention.^1^ Pelvic peritonitis is due to the disruption of the mucosal barrier of the genitourinary tract by either diseases or injuries resulting in spilling of microorganisms into the peritoneal cavity.^2^ Peritonitis is a common surgical and gynecological emergency with varying etiologies encountered worldwide such as: traumatic perforation of the uterus, septic abortion, puerperal sepsis, postoperative uterine infection, or endometritis complicating an intrauterine device, and gonococcal salpingitis.^2^ Its management poses diagnostic challenges in low-income countries (LIC); and it is further complicated by increase in antimicrobial resistance to predominant pathogens such as *E. coli, Klebsiella pneumoniae* and *Acinetobacter* spp.^3^ Early and definitive source control plus bacterial and toxins elimination reduces the need for re-operation and adverse outcomes including surgical site infection, fistula formation, hemorrhage, prolonged ileus, organ failure and death.^3^

Despite the fact that secondary peritonitis is one of the commonest cause of surgical and gynecological acute abdomen, few studies have been done regarding obstetric and gynecological-related peritonitis in LICs^4–6^ with each study addressing a single cause of pelvic peritonitis. Since majority (77%) of Tanzanians live in rural areas, the National Health Policy has set strategies to improve the health and wellbeing of all its inhabitants by upgrading health centers and scaling up training and use of non-physician clinicians to improve accessibility to comprehensive emergency obstetrical care (CEmOC) services in these areas.^7^ Hence the need to monitor the complications that might be associated with increased surgical interventions especially among cases from lower health facilities, due to the fact that there are increased referral pelvic peritonitis cases observed at BMC. Therefore, studying these cases to establish factors associated with outcomes and pathogens was critical for the purpose of initiation of appropriate management during admission in order to reduce associated morbidity and mortality. This study not only fills the pre-existing knowledge gap regarding the factors associated with short-term surgical outcomes among women presenting with pelvic peritonitis but also improves their prognosis and overall clinical outcome.

## METHODS

### Study Design, Duration, Population and Setting

This was a retrospective chart review of all patients with pelvic peritonitis from June 2019 to November 2021 followed by prospective study from December 2021 to May 2022. Both studies involved patients who were surgically managed for pelvic peritonitis at BMC, from the department of obstetrics and gynecological wards.

### Sample Size Estimation and Selection Criteria

A minimum sample size for this study was 101 participants, which was calculated using Yamane-Taro.^8^ All patients undergoing laparotomy for pelvic peritonitis in obstetrics and gynecological wards were enrolled in this study. The study excluded patients with pelvic peritonitis not originating from the female reproductive organs as determined intraoperatively, patients with primary peritonitis and patients with ruptured ectopic pregnancies.

### Sampling and Data Collection Methods and Tools

Retrospective and prospective data were collected using standardized data collection tool to capture clinical data (such as vital signs, symptoms), surgical (such as intraoperative findings, procedure performed) and laboratory data (FBP), culture and sensitivity and serum creatinine). In the retrospective arm, patients who had undergone laparotomy for peritonitis were identified from theatre registries; thereafter all respective files were retrieved from the medical records department. In the prospective arm, recruitment of patients was done at the emergency department and in the obstetrics and gynecological wards. Pelvic peritonitis was defined clinically by the presence of either abdominal muscle guarding, rigidity or rebound tenderness upon abdominal examination and confirmed by intraoperative findings due to the loss of integrity of the mucosa of the genital tract or structures.

In all participants enrolled prospectively, venipuncture was performed and approximately 4 mls and 2 mls of blood specimen were collected and placed in EDTA and plain vacutainer tubes for FBP and creatinine tests respectively. All samples were processed at BMC Central Pathology Laboratory which is accredited by the Southern African Development Community Accreditation Service (SADCAS) with a unique number MED 002 within 4 hours of specimen collection for processing. Aspirated specimen (pus/purulent and serosanguinous fluid) was obtained using a sterile syringe and transferred in a sterile specimen container and transferred to Catholic University of Health and Allied Sciences (CUHAS) Microbiology laboratory for bacteriological culture and sensitivity. In case of biopsy, the tissue was immersed in 10% neutral buffered formalin and processed as per protocol.^9^

### Laboratory Procedures Complete Blood Count and Creatinine Assays

Whole blood in EDTA vacutainer was used for complete blood count analysis using FBP Sysmex 1000 machine (Sysmex Corporation, Kobe, Japan) whereas, creatinine analysis was done using COBAS Integra 400 plus, (Roche Diagnostics Ltd, Rotkreuz, Switzerland).

### Culture and Sensitivity

Peritoneal pus and serosanguinous fluid specimen were processed as previously described^9^. Briefly, a 10 µL was used to directly inoculate sample on solid culture media including 5% sheep blood agar (BA; Oxoid, UK) and MacConkey agar (MCA; Oxoid, UK) plates and incubated at 35±2°C for 48 hours. After incubation, culture plates were evaluated for microbial growth and for positive growth microbial morphology (e.g., colony size and color) and characteristics (e.g., hemolysis on BA and lactose fermentation on MCA) were recorded.

Isolated bacteria were identified to possible species level by using conventional biochemical identification tests as reported previously^11^. Antibiotics susceptibility testing (AST) was performed by disk diffusion^12^ and interpreted using Clinical and Laboratory Standards Institute (CLSI) guidelines of 2021^13^. In this paper, isolates exhibiting intermediate activities against antibiotics were termed as resistant. *E. coli* ATCC 25922 and *S. aureus* ATCC 25923 were used as control strains to check the quality of culture media and antibiotic disks.

### Postoperative Follow Up Visit

Outcome variables in this study were determined as inhospital findings. Patients were visited by the Principal Investigator or Research Assistant on day one to look for presence of postoperative vomiting only, postoperative temperature change measured 48 hours after surgery, postoperative prolonged paralytic ileus assessed on day 4 post laparotomy and burst abdomen determined on day 8 post laparotomy. Postoperative control of laboratory investigations was done 48 hours after surgery: postoperative change in creatinine level was regarded as an increase in serum creatinine of ≥ 0.3 mg/dl (≥26.5mol/l) within 48 hours and postoperative change in hemoglobin level was regarded as a hemoglobin change of ≥ 3g/dl. Lastly, patients were followed to document post-operative complications and length of the hospital stay till day of discharge or death.

### Statistical Data Analysis

Data was entered into Microsoft excel for cleaning and coding, and then exported to Stata Statistical Software: Release 13. College Station, TX: StataCorp LLC for analysis according to the objectives of the study. The median (IQR) or mean (±SD) were calculated for continuous variables, whereas proportions and frequency tables were used for categorical variables. Bivariate analysis (Chi-square, χ^2^, and Fisher's exact tests) was done to test for the significance of association between independent variables and outcome variables. All factors with *P* value of ≤.2 were subjected to multivariable logistic regression analysis. Absence of post-operative complications, hospital stay ≤14 days and being alive on day 14 were considered as a good surgical outcome whereas death occurring within 14 days post-surgery or presence of any post-operative complications or prolonged hospital stay >4 days were regarded as a poor surgical outcome. A *P* value of <.05 at 95% confidence interval was considered to constitute a statistically significant difference.

### Ethical Considerations

Ethical clearance to conduct this study was sought from the Joint CUHAS/BMC Ethics and Review Committee before the commencement of the study with certificate number: CREC/511/2021. For the retrospective study, the administrative approval was obtained from the Bugando Medical Centre. Patients were requested to sign the written informed consent forms and were assured regarding confidentiality.

## RESULTS

### Socio-demographic Characteristics of Study Participants

A total of 101 participants were recruited in this study. The majority (79, 78%) of participants were reviewed retrospectively. The median [IQR] age of the study participants was 29 [24 to 35] years, ranging from 16 to 71 years. More than three quarters (77/101) were attended as referred patients. Approximately 23.8% of patients (24/101) developed pelvic peritonitis after being attended at BMC. Most of the patients (82.2%, 83/101) had pelvic peritonitis of obstetric in origin. Five patients developed pelvic peritonitis whilst pregnant. Only 11 (10.9%) patients had comorbidities and Human Immunodeficiency Virus (HIV) was the leading comorbidity (Table 1).

**TABLE 1:** Socio-Demographic Characteristics of Study Participants

Characteristic	Frequency (n)	Percentage (%)
Study period		
Retrospective	79	78.2
Prospective	22	21.8
Median age [IQR] in years	29	[24–35]
Referral status		
Referral	77	76.2
Non referrals	24	23.8
Referring facility		
Geita RRH	10	9.9
Sekou-Toure RRH	8	7.9
Othersa	59	58.4
Antecedent event		
Gynecology	18	17.8
Obstetrics	83	82.2
Reproductive profile		
Gravid	5	4.9
Non gravid	96	95.1
Comorbidity		
Present	11	10.9
Absent	90	89.1
Type of comorbidity		
DM	2	18.2
HIV	6	54.5
HTN	2	18.2
HTN & Cancer	1	9.1
HIV status		
Positive	6	5.9
Negative	63	62.4
Unknown	32	31.7

a Bariadi DH (n=2), Biharamulo CDH (n=4), Bukoba RRH (n=2), Bukombe DH (n=1), Buzuruga HC (n=2), CF Hospital (n=1), Shree Hindu Mandal Hospital (n=1), Igoma HC (n=3), Kakonko (n=1), Katavi RRH (n=1), Kirumba dispensary (n=1), Kitete (n=1), Magu DH (n=1), Maswa DH (n=2), Meatu (n=1), Misungwi DH (n=3), Musoma RRH (n=4), Nassa (n=1), Natta (n=3), Ngudu DH (n=3), Ngwalida (n=1), Nyamagana DH (n=5), Rubya Hospital (n=1), Sengerema DDH (n=1), Shinyanga RH (n=5) and Missing facility name (n=8).

### Clinical Profile of Study Participants

The median [IQR] duration of symptoms was 4 [3 to 7] days at enrollment. Abdominal pain was the most common symptom reported at 61.4%. The mean temperature and pulse rates were 37.7°C (±1.17) and 108 (±19.88) b/min respectively, all above normal values. More than half (58/97) of the study participants had elevated white blood cells. Severe anemia (hemoglobin <7g/dl) was recorded in 23.7% (23/97) of the participants. Low platelet count was seen in 21.6% (21/97) of the patients. Elevated creatinine levels were seen in 39.4% (28/71) (Table 2).

**TABLE 2:** Clinical Presentation of Study Participants Before Surgery

Variable	Frequency (n)	Percentage (%)
Late presentation		
>24 hours	82	81.2
<24 hours	19	18.8
Median duration of symptoms [IQR] in days	4 [3–7]	
Symptoms^a^ N=IOl		
Abdominal Pain	62	61.4
Abdominal distention	52	51.5
Fever	42	41.6
Abnormal vaginal discharge	18	17.8
Others	48	47.5
Vital signsa		
Temperature (SD) in degrees Celsius	37.7 (1.17)	
Pulse rate (SD) in beats/min	108 (19.88)	
Respiratory rates [IQR] in cycles/min	22 [19–34]	
SBP (SD) in mmHg	117.79 (22.02)	
GCS [IQR]	15 [15–15]	
Saturation [IQR] in %	97 [94–98]	
RBG [IQR] in mmol/1	6.1 [5.5–6.9]	
Required oxygen		
Yes	7	7.2
No	90	92.8
Missing^a^	4	
White blood cells		
High	58	59.8
Normal (4–11 × 109/1)	35	36.1
Low	4	4.1
Missing^a^	4	
Neutrophil % [IQR]	82.2 [73.6–87.6]	
Hemoglobin		
Normal (> 1 lg/dl)	14	14.4
Mild (10–10.9g/dl)	13	13.4
Moderate (7–9.9g/dl)	47	48.5
Severe (<7g/dl)	23	23.7
Missing^a^	4	
Platelets		
High	18	18.6
Normal (150–450xl03/mm^3^)	58	59.8
Low	21	21.6
Missing^a^	4	
Creatinine		
High	28	39.4
Normal (44–97μmol/L)	39	54.9
Low	4	5.6
Missing^a^	30	

a Removed from the denominator

### Intraoperative Characteristics

More than two-thirds (68/98) of the study participants developed pelvic peritonitis following caesarean deliveries. Of the study participants who had their American Society of Anaesthesiologists (ASA) classification recorded, 36.4% (32/88) had ASA 3 and above. Out of the 98 laparotomies that were performed, two-thirds (66/98) were first re-laparotomies. Junior doctors (residents and medical officers) performed 55.1% (54/98) of the surgeries. Necrotic and or dehiscent lower uterine segment was the most common intraoperative finding (40.8%). More than half of the study participants (54.1%) who underwent surgery had pus in their peritoneal cavities. Peritoneal cavity lavage was done to all patients followed by subtotal hysterectomy as the most prevalent procedure performed (69.4%). The median duration of surgery [IQR] was 110[80 to 150] minutes. Empirical postoperative antibiotics were prescribed to all patients with ciprofloxacin and metronidazole (41.8%) being the most frequent combination given. The median length of hospital stay [IQR] in days was 19 [7 to 35] (Table 3).

**TABLE 3:** Intraoperative Characteristics (N=98)

Variable	Frequency (n)	Percentage (%)
Preoperative diagnosis		
Post C/S peritonitis	68	69.4
Post SVD peritonitis	12	12.2
Gynecological peritonitis	18	18.4
ASA class		
1	13	14.8
2	43	48.9
3	27	30.7
4	2	2.3
5	3	3.4
Missing^a^	10	
Nature of laparotomy		
Primary	20	20.4
1st Relaparotomy	66	67.4
2nd Relaparotomy	11	11.2
3rd Relaparotomy	1	1
Surgeon Rank		
Junior	54	55.1
Senior	44	44.9
Intraoperative Findings		
Necrotic … I dehiscent LUS	40	40.8
Necrotic uterus	29	29.6
Pelvic abscess	6	6.1
Necrotic vault	4	4.1
Necrotic ovary	4	4.1
Foreign body	3	3.1
Visceral injuries	14	14.3
Pelvic seedling	1	1
Others^b^	93	94.9
Peritoneal fluid		
Pus	53	54.1
Serosanguinous	23	23.5
Feculent	3	3.1
Others^c^	14	14.3
Procedure performed		
Peritoneal lavage	98	100
STAH	68	69.4
Oophorectomy	6	6.1
Refreshing of vault margins	4	4.1
Visceral injury repair	10	10.2
Others^d^	121	123.5
Median duration of surgery [IQR] in minutes		110 [80–150]
Postoperative antibiotics		
CIP+MTZ	41	41.8
CRO-SUL+ MTZ	19	19.4
CRO+MTZ	16	16.3
Others^e^	22	22.5

a Removed from the denominator

b Necrotic fascia (n=4), Ruptured ovarian cyst (n=2), Endometrium (n=1), Ruptured uterus (n=7), TOA (n=3), Salpingitis (n=7), Oozing BTL site (n=1), Pyomyositis (n=1), Infected fibroid (n=1), Frozen pelvis (n=2), Uterine perforation (n=6), Lithopedion (n=1), Adhesions (n=30), RPOC (n=3), Placenta in peritoneum (n=4), Fetal parts in peritoneum (n=6) and Matted bowels (n=14).

c Blood (n=11), Urine (n=2), Pus … feculent (n=1).

d Refreshing of LUS margins (n=2), Salpingooophorectomy (n=4), Salpingectomy (n=2), Adhesiolysis (n=30), Foreign body removal (n=3), Removal of fetal parts (n=7), Marsupialization (n=1), Incision and drainage (n=1), Biopsy (n=3), Facial repair (n=2), Uterine repair (n=3), Myomectomy (n=1), Stoma placement (n=4), Drainage placement (n=56). Visceral repair (bowel anastomosis (n=4), gastric repair (n=1), bladder repair (n=2), ureter repair/reimplantation (n=3).

e Ampiclox (n=1), Ceftriaxone-sulbactam + ciprofloxacin +metronidazole (n=2), Ceftriaxone +ciprofloxacin+ gentamycin (n=1), Ceftriaxone + ciprofloxacin (n=1), Ciprofloxacin +metronidazole +gentamycin (n=14), Meropenem (n=1), Piperacillin-tazobactam + metronidazole (n=1) and Metronidazole+ gentamycin (n=1)

### Short-term Surgical Outcomes

Out of 101 study participants, 3 patients died before surgical intervention. Thus, surgery was performed in 98 patients only. Of these, 1 patient died within the same day, postoperative complications were reported in 37.1%(36/97) patients. Postoperative vomiting was the commonest complication reported. Nine patients had a fatal outcome (Table 4). Lastly, nearly three quarters (71/98) of the patients who underwent surgery had a poor surgical outcome (Table 4).

**TABLE 4:** Short-Term Surgical Outcomes

Variable	Frequency (n)	Percentage (%)
Postoperative complications (N=98)		
Yes	36	36.7
No	62	63.3
Postoperative vomiting on day 1 (N=96)	18	18.8
Postoperative temperature at 48 hours ≥ 380C (N=97)	6	6.2
Postoperative drop in Hb ≥3g/dl at 48 hours (N=23)	1	4.4
Postoperative rise in creatinine at 48 hours ≥26.5mol/l (N=22)	2	11.1
Postoperative prolonged paralytic ileus on day 4 (N=91)	12	13.2
Burst abdomen on day 8 (N=88)	12	13.6
Death (N=98)		
Died	9	9.2
Survived	89	90.8
Death within 24 hours	2	22.2
Death after 24 hours	7	77.8
Median length of hospital stay [IQR] in days		19 [7–35]
Duration hospital stay		
≤ 14 days	36	36.7
>14 days	62	63.3

### Factors Associated with Postoperative Complications

Of the several factors studied (age in years, referral, comorbidity, temperature >37.5°C, pulse rates >100b/min, respiratory rates >20cpm, systolic blood pressure (SBP) <90 mmHg, oxygen saturation <90%, duration of symptoms >24 hours, white blood cell (WBC) count >11x109/l, Hb ≤8g/dl, PLT <150 x103/mm3, rank of surgeon, laparotomy, ASA, necrotic &/dehiscent LUS), only having pulse rates >100 b/min (*P*=.041) and platelet counts <150 × 10^3^/mm^3^ (*P*=.049) were found to be statistically significant associated with developing postoperative complication. None of the factors were found significant in multivariable logistic regression (Table 5).

#### Factors Associated with Prolonged Hospital Stay

Being referred (*P*=.024), having preoperative body temperature of >37.5°C (*P*=<0.001), presenting to hospital more than 24 hours from onset of symptoms (*P*=.018), hemoglobin ≤ 8g/dl (*P*=.003), number of relaparotomies (*P*=.001), having a necrotic and or dehiscent LUS (*P*=.02) and operative time >120 minutes (*P*=.012) were significantly associated with a prolonged hospital stay (Table 5). Only elevated temperature (aOR, 5.08; 95% CI 1.23 to 20.97; *P*=.025) remained significant on multivariable logistic regression.

### Factors Associated with Death

Of the several factors studied (age in years, referral, comorbidity, temperature >37.5°, pulse rates >100b/min, respiratory rates >20cpm, SBP <90 mmHg, saturation <90%, duration of symptoms >24 hours, WBC count >11x109/l, Hb ≤8g/dl, PLT <150 x 103/mm3, rank of surgeon, laparotomy, ASA, necrotic &/dehiscent LUS), only ASA (*P*=.045) was found to be associated with death in this study (Table 5).

**TABLE 5:** Multivariable Analysis of Factors Associated with Short-Term Surgical Outcomes

Variable	Yes	No	Chi2 (x^2^)/Fishers exact	P Value	aORa [95% CI]	P Value
**Analysis: 1 Postoperative complication**						
Pulse rates > 100b/min						
Yes	27	33	4.191	.041	2.14 [0.80–5.96]	.129
No	9	28	1			
PLT < 150 x103/mm3						
Yes	11	10	3.882	.049	2.16 [0.77–6.08]	.143
No	21	51	1			
**Analysis: 2 Prolonged hospital stay**						
Referral						
Yes	53	22	5.063	.024	2.88 [0.59–13.96]	.189
No	10	13	1			
Temperature > 37.50C						
Yes	38	8	12.12	<.001	5.08 [1.23–20.97]	.025
No	21	23	1			
Duration of symptoms > 24 hours						
Yes	56	24	5.638	.018	0.96 [0.17–5.54]	.967
No	7	11	1			
Hb ≤ 8g/dl						
Yes	36	11	8.824	.003	1.81 [0.42–7.81]	.425
No	23	24	1			
Relaparotomy						
1st	50	16		<.001	3.72 [0.43–32.18]	.232
2nd	7	4			1.2 [0.06–23.43]	.906
3rd	1	0				
Necrotic &/dehiscent LUS						
Yes	31	9	5.416	.02	1.58 [0.32–7.90]	.578
No	25	20	1			
Duration of surgery > 120 minutes						
Yes	27	7	6.294	.012	1.78 [0.36–8.68]	.478
No	29	24	1			
**Analysis:3 Death**						
ASA						
1	2	11		.045		
2	2	41			0.23 [0.03–1.91]	.173
3	3	24			0.53 [0.07–4.03]	.537
4	0	2			Omitted	
5	2	1			5.65 [0.25–128.11]	.277

a Multivariable regression analysis

### Patterns of Bacteria and their Drug Susceptibility

A total of 25 patients, 10 retrospectively and 15 prospectively, had culture and sensitivity results. Out of 25 samples, 23 had positive microbial growth from which 27 bacterial pathogens were isolated. The predominant pathogen was Gram negative bacteria with *E. coli* (7/23) and *K. pneumoniae* (4/23) being frequently isolated.

**FIGURE 1:**
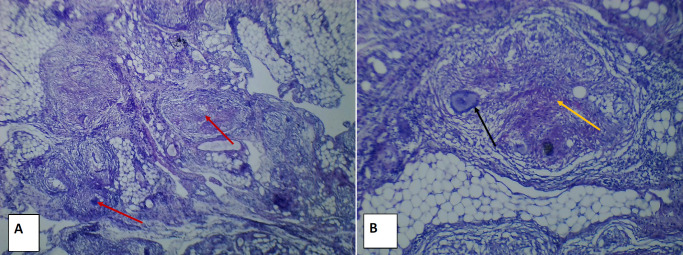
Hematoxylin and Eosin Tissue Stain Showing Section of Omental Tissue Fragments

Moreover, a biopsy of one patient revealed features of *Mycobacterium tuberculosis* infection of the pelvic structures (Figure 1).

Hematoxylin and eosin tissue stain showing section of omental tissue fragments of multiple granuloma lesion pointed by red arrow in diagram A above (x10) hpf. Multinucleated giant cell of Langerhans cells type pointed by black arrow and central caseous necrosis pointed by yellow arrow in diagram B (x10) hpf

Other bacteria isolated were *Acinetobacter* spp. (n=2), *Citrobacter freundii, Citrobacter* spp., Coagulase negative *S. aureus* (n=2), *Edwardsiella tarda, Enterobacter* spp., *Enterococcus* spp., *S. aureus*, *S. pyogenes*, *Proteus vulgaris* and significant growth of unidentified organism.

### Drug susceptibility Profile of the 27 isolates from patients with pelvic peritonitis

Gram-positive bacterial pathogens were more sensitive to gentamicin (75%) and linezolid (75%) and less sensitive to erythromycin (25%), (Table 6). Gram-negative bacterial isolates exhibited more resistance towards penicillins (amoxicillin-clavulanic acid and ampicillin, respectively), ciprofloxacin and 3^rd^ generation cephalosporins and they were less resistance to amikacin 13.3% and no resistance to meropenem was observed (Table 7).

**TABLE 6:** Antibiotics Susceptibility Testing of Gram-Positive Bacteria

NO	Microbial Isolate	CN	CIP	OXA	SXT	ERY	CD	VAN	LZD	TET	MDR
013	*S. aureus*	S	S	S	S	S	S	NT	S	NT	No
2330	*S. epidermidis*	S	S	NA	S	R	R	S	S	NT	No
2404	*S. pyogenes*	S	R	NA	NT	R	R	R	S	R	Yes
014	*Enterococcus spp.*	R	R	NA	NT	R	NT	R	R	S	Yes

Abbreviations: CIP, ciprofloxacin; CLI, clindamycin; ERY, erythromycin; CN, gentamycin; LZD, linezolid; NA, not applicable; SXT, trimethoprim-sulfamethoxazole; TET, tetracycline; OXA, oxacillin; and VAN, vancomycin; NT, Not tested.

**TABLE 7:** Antibiotics Susceptibility Testing of Gram-Negative Bacteria

NO	Microbial Isolate	AMP	CRO	CN	ClP	AK	MEM	TZP	SXT	AMC	FEP	CAZ	CTX	MDR
OOl	*E.coli*	R	S	S	R	NT	S	S	R	S	S	S	NT	Yes
002	*E.coli*	R	R	NT	R	S	S	NT	R	R	R	NT	NT	Yes
003	*E.coli*	R	R	NT	NT	S	S	R	R	NT	R	NT	NT	Yes
004	*E.coli*	R	R	NT	NT	S	S	R	R	NT	R	NT	NT	Yes
006	*E.coli*	R	R	R	NT	R	S	NT	R	R	NT	R	NT	Yes
007	*E.coli*	R	R	NT	R	NT	NT	S	NT	R	NT	NT	NT	Yes
008	*E.coli*	NT	R	R	R	S	NT	NT	R	R	NT	R	NT	Yes
009	*K.pneumoniae*	NT	R	R	R	S	S	NT	NT	S	NT	R	NT	Yes
005	*K.pneumoniae*	R	R	R	R	S	NT	NT	R	R	NT	R	NT	Yes
2236	K.pneumomae	NT	R	NT	NT	S	S	S	NT	S	NT	R	NT	No
003	*K.pneumoniae*	R	R	NT	NT	S	S	R	R	NT	R	NT	NT	Yes
011	*P.aeruginosa*	NT	NT	R	NT	NT	S	S	NT	NT	NT	R	NT	No
012	*P.aeruginosa*	NT	NT	NT	R	S	S	S	NT	NT	S	S	NT	No
015	*P.aeruginosa*	NT	R	R	R	S	NT	NT	R	R	NT	R	NT	Yes
016	*Citrobacter freundii*	NT	R	R	S	NT	S	NT	R	NT	NT	NT	R	Yes
017	*Citrobacter spp.*	R	R	R	S	R	S	NT	NT	R	R	R	R	Yes
018	*Acinetobacter spp.*	NT	R	NT	R	S	S	R	R	NT	NT	R	NT	Yes
019	*Acinetobacter spp.*	R	R	R	R	NT	S	R	R	R	R	R	NT	Yes
020	*Enterobacter spp.*	NT	R	S	S	NT	S	NT	NT	NT	R	R	R	No
021	*Proteus vulgaris*	NT	NT	NT	S	NT	NT	NT	NT	NT	NT	NT	NT	N/A
022	*Edwardsiella tarda*	NT	S	NT	R	NT	NT	R	NT	NT	S	S	S	N/A
010	Unidentified	NT	NT	NT	NT	S	S	R	NT	R	R	R	NT	No
023	Negative bacilli	NT	R	R	R	S	S	NT	NT	S	NT	R	NT	Yes

Abbreviations: AMP, ampicillin; AK, amikacin, CRO-ceftriaxone; CIP, ciprofloxacin; CN, gentamycin; MEM, meropenem; TZP, piperacillin-tazobactam; SXT, trimethoprim-sulfamethoxazole; AMC, amoxicillin-clavulanic acid; FEP, cefepime; CAZ, ceftazidime; CTX, Cefotaxime; NT, Not Tested.

## DISCUSSION

The study observed that most of the cases of pelvic peritonitis were obstetric in origin, with a history of caesarean section as a major antecedent event. Furthermore, it was observed that multi-drug resistant gram-negative bacteria were the most frequently isolated pathogens. Various factors such as elevated preoperative body temperatures and low platelets were found to be associated with post-operative complications including prolonged hospital stay.

The observation of cesarean section as the major event resulted to peritonitis, could be attributed to the increased number of CEmOC centers country wide that offer surgical services as evidenced by the fact that the majority of cases attended at BMC were referrals.^7^ Tanzania needs to expand the number of facilities providing these services in more remote areas. Considering severe shortage of human resources for health in the country, currently operating at 32% of the required skilled workforce, an intensive three-month course was developed to train non-physician clinicians for remote health centres. Methods: Competency-based curricula for assistant medical officers' (AMOs These CEmOC centers need to be further assessed to ensure infection, prevention and control (IPC) guidelines are adhered to as recommended by the Ministry of Health (MoH).^14^ Majority of the study population presented at a median age of 29 years; this is due to the fact that the participants were at their reproductive age.

Comorbidity was not statistically associated with the development of surgical complications. The small sample size could account for this. However, amongst those with comorbid conditions, death occurred only in patients with HIV. A competent immune system helps to contain and clear infections as opposed to being immunocompromised. Comorbidity among patients with peritonitis in Rwanda and BMC^15^ was also found to be a predictor of mortality whereby in Rwanda, those with any comorbidity were also at risk of dying as compared to those without any comorbidity.^16^

Majority of participants were referred, a late presentation to hospital amongst our study participants was a key feature; 81.2% of patients came more than 24 hours of onset of symptoms; with a median duration of symptoms of 4[3 to 7] days similar to what was observed in in Rwanda.^16^

The commonest intraoperative finding in this study population was a necrotic and or dehiscent LUS followed by necrotic uterus implying that the source of infection is the uterus. Ascending infections from the lower genital tract may cause microbial colonization of lower uterine segment incisions. Prolonged rupture of membranes and multiple vaginal examinations during labor assessment could contribute to this observations.^16^ Also, spillage of infected amniotic fluid into the peritoneal cavity as in the case of chorioamnionitis or during extraction of a macerated baby can serve as a direct inoculation of the uterine incision or cavity by any microorganism present. Subtotal abdominal hysterectomy emerged as the predominant procedure performed in these patients. Eliminating the source of infection rather than a more conservative approach was more practical considering the extent of infection and the limited choice of antibiotics due to increase in antimicrobial resistance (AMR) in our setting.^16^ Drainage facilitated removal of any pathological peritoneal fluid that was either gross or obvious or minimally occult in areas not easily visible by the surgeon's eye. Despite this intervention in our study participants, it was noted that 16.3% of the patients who received this modality required relaparotomies of which 68.8% (11/16) were due to persistent infection. More research is needed to come up with the evidence regarding the lavage and drainage insertion to ensure standardized treatment guidelines.

Postoperative complications such as burst abdomen, postoperative prolonged paralytic ileus and postoperative vomiting among others were reported in 36.7% of patients who had pelvic peritonitis. This was comparable to a previous study done at BMC seven years ago.^14^ A case fatality rate of 9.2% was observed in this study and was lower than that reported in previous studies.^14,15^ This may be due to the advances in provision of medical and surgical services. In addition, fewer visceral injuries namely gastric and bowel perforations were found in our study population compared to those reported in Mabewa's study.^14^

The median hospital stay was 19[IQR:7 to 35] days in this study, it was more than that observed in a previous study^14^ due to the fact that 12 of our patients had burst abdomen that necessitated relaparotomies. In this study, those who underwent a 1^st^ relaparotomy had an almost 4-fold risk of a prolonged hospital stay. Relaparotomies impacts the health of patients negatively such as anemia, surgical site infections and poor nutrition status.^18^

Gram negative bacteria were the predominant pathogens in the cultured specimen; with *E. coli* being the most prevalent isolated microorganism followed by *K. pneumoniae*. These findings were also reported by Halfon in Rwanda ^16^ and among women with puerperal sepsis by Alcard.^17^ Despite BMC having culture and sensitivity services, the utilization of the laboratory for this service was very low as evidenced by the fact that only 14.9% of the retrospective patients had culture and sensitivity done despite pus being detected intraoperative. This translates that many clinicians in our setting and other settings in Tanzania do not routinely order culture and sensitivity tests as a standard of care and practice to ensure targeted antibiotic prescription on a patient suffering infection.

In this study, it was observed that majority of gram-negative bacteria were MDR. This could be contributed to misuse of the antibiotics in the health care system.^19^ The majority of Gram-negative bacteria were resistant to third generation cephalosporins, gentamycin and ciprofloxacin and this was almost 3-fold higher (32%) than that reported among cultured isolates in Rwanda.^16^ These data justify the need to review the current regime used as surgical prophylaxis in our setting.^17^ As observed in many other studies conducted in our setting, all of our isolates were still sensitive to meropenem.^17,18,21^

### Study limitations

The main limitations of this study were: anaerobic cultures were not performed, this could underestimate the role of anaerobes; and as for the retrospective aspect of the study, some important values such as vital signs, laboratory investigations and culture and sensitivities were either not recorded or collected making it hard to have a large sample size for some variables.

## CONCLUSION

Pelvic peritonitis as a result of caesarean section carries significant morbidity and mortality. Nearly three quarter of patients end up with poor surgical outcomes. MDR gram-negative bacteria were the predominant microorganisms causing pelvic peritonitis. There is a need of timely complete sepsis work-up (screening of sepsis markers and culture and sensitivity) of the patients with peritonitis to ensure appropriate management and timely individualized antibiotic treatment to prevent associated morbidity and mortality. Furthermore, there is a need to conduct a clinical audit on the quality of caesarean sections coupled with continuous mentorship to improve competencies of health care staff in CEmOC centers and other regional and district hospitals. Lastly, prospective study with a large sample size with a good sepsis work-up and culture and sensitivity is needed to ensure comprehensive data are collected to confirm observations of this study to guide establishment of local guidelines of managing pelvic peritonitis.
